# The effectiveness of group problem-solving therapy on women's sexual function and satisfaction after mastectomy surgery

**DOI:** 10.1186/s12905-022-01628-x

**Published:** 2022-02-23

**Authors:** Mahshid Bokaie, Ommolbanin Firouzabadi, Azadeh Joulaee

**Affiliations:** 1 Research Center for Nursing and Midwifery Care, Shahid Sadoughi University of Medical Sciences, Yazd, Iran; 2 Shahid Sadoughi University of Medical Sciences, Yazd, Iran; 3 Special Breast Unit, Mahdieh University Women’s Hospital, Shahid Beheshti University of Medical Sciences, Tehran, Iran

**Keywords:** Breast neoplasms, Mastectomy, Sexual dysfunction, Sexual satisfaction, Problem-solving, Women

## Abstract

**Background:**

Breast cancer is the second cause of death and the most common cancer in women worldwide, threatening different aspects of individual and mental health, quality of life, sexual function, and sexual satisfaction. This study aimed to determine the effectiveness of group counseling based on a problem-solving solution on women's sexual function and satisfaction after mastectomy surgery.

**Methods:**

The present research was an open pilot study, with a pretest, a post-test, and a follow-up period. Of women referred to the Tehran Breast Cancer Institute, 32 were selected using convenience sampling. The group received eight 90-min sessions of problem-solving solution counseling. This approach is based on cognitive-behavioral therapy and can improve an individual's ability to cope with stressful life experiences. The data collection tool was FSFI and sexual satisfaction questionnaires, which were filled before the intervention (baseline), immediately after the intervention, and one month later (follow up). Data analysis was performed using SPSS 21 statistical software application at the certainty level of 95% (*P* < 0.05).

**Results:**

The mean FSFI score increased from 18.37 ± 8.35 before the intervention to 20.88 ± 7.67 immediately after the intervention and 22.95 ± 5.79 one month later (*P* < 0.0001). Also, the mean sexual satisfaction score was 65.27 ± 5.98, 68.08 ± 5.61, and 70.46 ± 5.35 before the intervention, immediately after the intervention, and one month later, respectively (*P* < 0.05). The results also showed that although the two components of sexual function and satisfaction were statistically significant after sexual counseling, this improvement was not clinically progressive. The mean sexual function and satisfaction score was still low after sexual counseling.

**Conclusions:**

It was observed that sexual function and satisfaction were improving among the patients after the intervention. Thus, sexual health counseling sessions are recommended for breast cancer patients.

## Background

Breast cancer constitutes almost one-fourth of all cancers in women [1], and it is the most common cancer among them. One out of eight women is suffering from breast cancer, afflicting almost 1.5 million women each year. Moreover, women dying from breast cancer stand at the top of the mortality list. The incidence of breast cancer in Iran is reported as 31 per 100,000 women, and the cancer is most prevalent in the 42–49 age group [2]. Breast cancer is the most common type of cancer (fifth in cancer mortality) in Iranian women [3]. It affects both physical and mental dimensions in an individual [4]. In most societies and cultures, the breast is considered a female organ, and because of its importance in forming the female identity [5], reaction to breast cancer can include fear, anxiety, and depression. Some of the new causes of these problems create implicit meanings of this diagnosis in the patient's mind including the likelihood of body deformation, pain, lack of financial and social support, losing female identity and sexual desire, reduced social activity, concern about ambiguous future, recurrence of disease, and death [6].

Iranian women with breast cancer greatly focus on their maternal role, have a great emotional involvement with their children, and effectively cope with their maternal role. Moreover, in the Iranian culture, the wife and the maternal role significantly contribute to women's identity [7].

In addition, Iranian women feel great commitment to fulfill their husbands’ sexual needs to protect their families and therefore attach great value to their husbands’ patience for their probable sexual problems [8].

Female sexual dysfunction (FSD) is increasingly being identified as a problem worldwide. Women can have problems in various parts of the sexual cycle including desire, arousal, lubrication, orgasm, satisfaction, or pain experience related to sexual activity [9]. Sexual changes are associated with emotional consequences, feeling unattractive or lacking femininity, and concerns about their impact on the partner or relationship [10]. Sexual dysfunction possibly remains more than one year after breast cancer diagnosis. It may be that chemotherapy is the cause of all sexual problems such as reduced sexual desire and mental arousal, vaginal dryness, and dyspareunia [11]. It seems that mastectomy directly impacts the sexual function of women [12].

Sexual satisfaction is a multi-component concept including emotional and physiological aspects of sexual life. Sexual satisfaction is not limited to physical joy, and it includes all positive and negative emotions after sexual intercourse [13]. Providing counseling services for patients reduces their tension so that they consider others’ counsels and assistance vital for compatibility with their emotions [14]. Group counseling is a two-sided process in which the counselor and the identical group study problems and approaches. In a group counseling environment, members show a large degree of reaction toward each other, which increases insight acquisition. Counseling programs can improve sexual function and satisfaction [15]. Problem-solving treatment has been used to help people with cancer generate and evaluate various solutions for challenges they face in life [16].

Hummel et al. [17] designed internet-based cognitive behavioral therapy to improve sexual dysfunctions in women treated for breast cancer.

Educating the problem-solving issue mentions a cognitive-behavioral process that provides a variety of alternative and potential responses for controlling the problematic conditions and increases the possibility of choosing the best and most effective alternative responses. The advantage of the problem-solving method is that we can use it for individual and/or group treatments. This approach is based on cognitive-behavioral therapy and could improve an individual's ability to cope with stressful life experiences [18]. This type of treatment will initiate active cooperation between the patient and the therapist [19]. Problem-solving treatment is a brief, structured psychological intervention. The treatment shares with other cognitive-behavioral treatments a focus on here and now rather than dwelling on past experiences and regrets. The treatment involves active collaboration between the patient and the therapist, with the patient taking an increasingly active role in planning treatment and implementing activities between treatment sessions [20].

### Aim of the study

This study aimed to determine the effectiveness of group counseling based on a problem-solving solution on women's sexual function and satisfaction after mastectomy surgery.

## Methods

### Design and setting

The present research was a semi-experimental study, with a pretest, a post-test, and a one-month follow-up period. Written informed consent was obtained from all the participants. The patients were contacted based on the list available at the institute and according to the admission criteria. The samples were selected based on the inclusion criteria by reviewing the existing files. Breast cancer patients can choose this institution for treatment and use its services. Of 40 patients referred to the Tehran Breast Cancer Institute between Aug and Dec 2020, 32 were invited to participate in the study according to the inclusion and exclusion criteria. The sample size formula was ($${\varvec{N}} = \frac{{2\user2{*}\left( {{\varvec{Z}}_{{\left( {1 - \frac{ \propto }{2}} \right)}} + {\varvec{Z}}_{{(1 - {\varvec{\beta}})}} } \right)^{2} \user2{*p*q}}}{{({\varvec{p}}_{0} - {\varvec{p}}_{1} )^{2} }}$$ where $${\varvec{p}} = \frac{{{\varvec{p}}_{0} - {\varvec{p}}_{1} }}{2}$$, $${\varvec{q}} = 1 - {\varvec{p}}$$, with %95 confidence interval, %5 alpha, and %15 chance of falling). The students were assured that the study results were confidential and would be published without names. They could leave the research at any stage.

### Eligibility criteria

The inclusion criteria were as follows: cancer stages between I and III, 1–5 years passing from breast surgery, Iranian nationality, residence in Tehran, 30–59 years of age, ability to read and write, mastectomy, being married, having a single partner, not participating in any other consulting class, and finished chemotherapy.

The exclusion criteria were lumpectomy surgery, cancer recurrence, history of significant physical and mental illnesses such as schizophrenia and major depression, and drug abuse.

### Instruments

The research instruments included a demographic characteristics questionnaire, the female sexual function index (FSFI) questionnaire [21], and the Larson sexual satisfaction questionnaire [22]. The demographic characteristics questionnaire included age, education, marital status, contraceptive method, breast cancer grading, type of surgery, chemotherapy, and radiotherapy records.

The female sexual function index (FSFI) includes 19 questions, each with four answer options. This standard questionnaire measures six dimensions of sexual function (sexual desire (two items), sexual arousal (four items), lubrication (four items), orgasm (three items), sexual satisfaction (three items), and pain (three items) over the recent four weeks. Measures were taken as per the questionnaire to determine each person's score in each section and determine the overall score. The lowest score of 2 is the maximum of 36. The cutting score for determining sexual disorders is 26 or less. The total point obtained is calculated in different areas. The total score is obtained by adding up six sections Brown, 2000). The questionnaire was validated by Fakhri et al. The general test–retest reliability coefficients were acceptable for each domain of the questionnaire (from 0.73 to 0.86) and the internal consistencies (from 0.72 to 0.90) [24].

The sexual satisfaction questionnaire is the Iranian version of the sexual satisfaction questionnaire consisting of 25 5-answer questions. The items in the questionnaire are scored based on a Likert scale from one to five, with "never" receiving 1, "rarely" receiving 2, "sometimes" receiving 3, "most of the times" receiving 4, and "always" receiving 5. A total score of 25–75 is equal to low sexual satisfaction, 76–100 to medium sexual satisfaction, and 101–125 to high sexual satisfaction. Reliability was determined using Cronbach's alpha coefficient (0.8 for positive question and 0.77 for negative one) and intra-class correlation coefficients (ICC = 0.8) (23, 24).

### Interventions

This study was accomplished in fall and winter 2020. After obtaining informed consent, the participants provided their phone numbers and addresses to participate in the study. The time, date, and place of the counseling sessions were announced by phone. A counseling program based on a problem-solving approach, including eight 90-min counseling sessions per week, was designed for the participants based on a review of texts and the research team's opinions. The participants formed four groups, each consisting of eight individuals in a suitable location in the breast cancer institute. A summary of the contents of the sessions is outlined in Table 1. The questionnaires were completed by the participants before the intervention (baseline), immediately after the intervention, and one month later (follow up).

**Table 1:** Subjects of discussions in each counseling session

Sessions	Subjects of discussion
One	The introduction, anatomy, and physiology of the reproductive system, the sexual cycle, the female sexual organ and erogenous zones and factors affecting them, explanation on how to fill in the questionnaire, and presenting home assignments
Two	The importance of sexual relation and the lack of concentration on penetration in all sexual relations of the couple, speaking about cancer and its effects, treatments, and complications, presenting homework, and explanation on how couple get along with this problem
Three	Training problem-solving skills, confronting with stress, techniques of relaxation and establishing effective communication, exchange of views regarding alternative solutions for improving the couple’s relation in breast cancer, giving home assignments
Four	The review of sexual schemas and women’s concerns and their control, review of their sexual expectations, training sexual skills, sensational exercises, necessary readiness for relation, explanation regarding home assignments such as common massage, and giving home assignments
Five	Free discussion regarding issues and problems, defining exact conformation of the issue, determining a range of possible solutions, analyzing solutions, problems created following the execution of individual solutions, weaknesses, and strengths of individual solutions, anticipating consequences, developing and strengthening communication skills, managing negative excitement (negative anger and excitement), training relaxation, responding to questions, and giving home assignments
Six	Providing suitable solutions against complications caused by cancer in sexual matters, changes in appearance, improving communication techniques, reducing worries through counseling, providing introduction to the Kegel exercise and other effective factors in strengthening the sexual function (nutrition, concentration on moments of enjoying it), responding to questions, and giving home assignments
Seven	The review of previous sessions and implementing a solution for real cases set forth
Eight	The presence of spouses and study of their sexual expectations, talking about sexual and mental differences of men and women in each stage of the sexual cycle, sexual interactions, and sexual response and solutions to improve sexual satisfaction, explaining on how to fill in the questionnaire

### Ethical considerations

The study was performed under the Declaration of Helsinki and approved by the Ethics Committee of the Research Deputy of the Shahid Sadoughi University of Medical Sciences (code: IR.SSU.MEDICINE.REC.1397.176).

After explaining the study's aims for the participants, written informed consent was obtained from all of the participants. Confidentiality was ensured.

### Data analysis

The data were analyzed using descriptive statistics and inferential statistics via SPSS 21 software (SPSS, Inc., Chicago, IL, USA). A significant value was considered less than 0.05. Since the distributions of the studied variables were normal, parametric statistical tests, such as variance analysis used, repeated measures, and the Bonferroni post hoc test, were performed.

The training of the five problem-solving skills began based on the problem-solving steps of Dezorella and Goldfried. The steps included defining and planning the problem, analyzing the problem, determining real goals, producing a solution, deciding and choosing the best solution, predicting possible consequences of each action, paying attention to the usefulness of these consequences, implementing the selected solution, and reviewing and evaluating the steps of problem-solving skills.

Sample assignments:

- A list of symptoms and complications of treatment was prepared and shared in the next consultation
- Preparing a list of ways to deal with negative thoughts and strengthen positive thoughts, reviewing solutions
- Practicing problem-solving skills (reducing intimacy and fear of having sex) and practicing strategies and suggestions in the following counseling session
- Practicing anger management strategies, reducing negative emotions, practicing active listening, and sharing results in the following counseling session
- Dedicating time and space to oneself, practicing relaxation skills, practicing sensory exercises, breathing training, practicing joint massage, and Kegel exercising and sharing it with one’s spouse

Due to the extent of the problem in counseling sessions, the problem-solving and cognitive-behavioral methods became a component.

## Results

This study was performed on 32 women after mastectomy referred to the Tehran Breast Cancer Institute. The average age of the women was 39.81 ± 7.54, the average spouse’s age was 43.81 ± 8.67, the marriage duration average was 14.9 ± 81.59, the cancer affliction period was 4.69 ± 3.08, and the post-treatment interval was 3.47 ± 3.12. All the participants experienced chemotherapy, and 27 (84.4%) of them also experienced radiotherapy. Some of the demographic characteristics are mentioned in Table 2.

**Table 2:** The descriptive characteristics of the women after mastectomy surgery

Variable	Number	Percentage
*Women education*		
Under diploma	6	18.8
Diploma	11	34.4
University degrees	15	46.8
*Spouse education*		
Under diploma	6	18.8
Diploma	11	34.4
University degrees	15	46.8
*Women*^*'*^*s occupation*		
Housewife	20	62.5
Employed	12	37.5
*Spouse’s occupation*		
Employee	10	31.2
Self-employed	3	9.4
Laborer	19	59.4
*Contraception method*		
Hormonal	3	9.4
IUD	7	21.8
Withdrawal	18	56.3
TL	4	12.5
*Cancer grading*		
I	6	18.8
II	21	65.6
III	4	12.5
IV	1	3.1

The mean sexual satisfaction score was 65.27 ± 5.98, 68.08 ± 5.61, and 70.46 ± 5.35 before the intervention, immediately after the intervention, and one month later, respectively. Table 3 shows the statistical test of a significant difference in the three periods (*P* < 0.05).

**Table 3:** The mean difference of sexual satisfaction in three periods

Time	Mean ± SD	Time	Mean ± SD	P^*^
Before intervention (baseline)	65.27 ± 5.98	Immediately after intervention	68.08 ± 5.61	0.02
Immediately after intervention	68.08 ± 5.61	One month later (follow-up)	70.46 ± 5.35	0.03
One month later (follow-up)	70.46 ± 5.35	before Intervention (baseline)	65.27 ± 5.98	0.008

*P < 0.05 shows the statistical test of a significant difference in the three periods

The mean FSFI score increased from 18.37 ± 8.35 before the intervention to 20.88 ± 7.67 immediately after the intervention () and 22.95 ± 5.79 one month later (follow up) (*P* < 0.0001). The mean score of the six-fold dimensions of sexual function is shown in Table 4. Table 5 compares the mean total score of sexual function in the two periods using the Bonferroni test.

**Table 4:** Comparison of the mean total score of sexual function and its dimensions in three periods

Dimensions of FSFI	Before intervention (baseline) (Mean ± SD)	Immediately after intervention (Mean ± SD)	One month after intervention (follow-up) (Mean ± SD)	F	P^*^
Total score	18.37 ± 8.35	20.88 ± 7.67	22.95 ± 5.79	20.53	< 0.001
Sexual desire	2.98 ± 0.67	2.99 ± 0.68	3.11 ± 0.71	15.96	< 0.001
Sexual arousal	2.98 ± 1.30	3.22 ± 1.04	4.43 ± 0.98	6.98	< 0.003
Lubrication	2.91 ± 1.47	3.36 ± 1.32	4.02 ± 0.99	24.02	< 0.001
Orgasm	3.24 ± 1.78	3.81 ± 1.36	4.08 ± 0.96	10.39	< 0.001
Satisfaction	3.39 ± 1.67	3.90 ± 1.54	4.07 ± 1.02	11.63	< 0.001
Pain	3.18 ± 2.08	3.60 ± 1.73	3.24 ± 1.14	10.51	< 0.001

*P < 0.05 shows the statistical test of a significant difference in the three periods

**Table 5:** Comparison of the mean total score of sexual function in two periods using the Bonferroni test

Time	Mean ± SD	Time	Mean ± SD	P^*^
Before intervention (baseline)	18.37 ± 8.35	Immediately after intervention	20.88 ± 7.67	< 0.001
Immediately after intervention	20.88 ± 7.67	One month later (follow-up)	22.95 ± 5.79	< 0.001
One month Later (follow-up)	22.95 ± 5.79	Before intervention (baseline)	18.37 ± 8.35	< 0.001

*P < 0.05 shows the statistical test of a significant difference in the three periods

## Discussion

This study aimed to determine the effectiveness of group counseling based on problem-solving solutions on women's sexual function and satisfaction after mastectomy surgery.

Other findings of the present study indicated that group counseling based on problem-solving solutions had no clinical effect on women's sexual function and satisfaction after mastectomy surgery.

Numerous studies have been conducted on psychological counseling issues in women with breast cancer [27, 28]. However, few studies have been performed on the effect of sexual health counseling on women with breast cancer. Studies carried out in different countries have repeatedly stated that women feel that their sexual function is affected by mastectomy.

Molavi et al. [29] evaluated couples' sexual function and satisfaction after mastectomy surgery. In this study, the mean FSFI score was estimated in mastectomy patients, and most of them reported moderate sexual satisfaction.

Many studies addressed the sexual problems of women with breast cancer. In a study conducted by Kedde et al. [30], young women undergoing breast cancer treatment had a more negative experience about sexuality and were less sexually active [30]. Zahedian et al. [31] showed that group meta-cognitive therapy improved depression in women with breast cancer.

Hamzehgardeshi et al. [32] showed the effect of midwifery-based counseling support programs on the body image of breast cancer women survivors.

The mean FSFI score of mastectomy patients in their study is in line with that in our research. However, the mean sexual satisfaction score in our study is lower than that in their study. Reese et al. [33] showed that sexual concerns for breast cancer survivors were not mostly removed, and evidence-based interventions were necessary, particularly in cases that the survivors had partners [33]. This study confirms the participation of couples in the intervention that we also proposed.

Fatehi et al. [34] showed that the psychosexual program effectively improved sexual function and quality of sexual life among Iranian breast cancer survivors.

A recent study is observed to be in line with our study regarding women's sexual function. In this study, although FSFI was statistically significant after the intervention, sexual function did not overtake from the cut off point. Sexual satisfaction in our study statistically significantly improved from 65.27 ± 5.98 to 68.08 ± 5.61. However, Fatehi et al. reported no change in the statistical and clinical dimensions.

Combining problem-solving with another method and continuing group counseling sessions seem to be highly effective.

We were not able to include couples in the study due to limitations. Studies on interventions performed on couples are highly effective. In our opinion, if, after the diagnosis of cancer, step-by-step counseling sessions are performed simultaneously with the treatment steps and according to the individual's needs, the intervention will be highly effective and efficient.

### Study limitations

None of the participants wanted to be in the control group. Therefore, this study was not a randomized clinical trial. Also, many women after mastectomy surgery were not members of the Tehran Breast Cancer Center. Moreover, due to cultural reasons, meetings were not held for the spouses, and couple therapy is recommended for these patients. The short-term follow-up period (one month) was another limitation of this study.

## Conclusion

The present study results showed that although the two components of sexual function and satisfaction were statistically significant after sexual counseling, this improvement was not clinically progressive. The mean sexual function and satisfaction score was still low after sexual counseling. As sexual function and satisfaction improve, continuing sexual health counseling sessions for breast cancer patients is recommended.

## Data Availability

The datasets used and analyzed during the present study are available from the corresponding author on reasonable request.

## Abbreviations

FSFI: The Female Sexual Function Index
FSD: Female Sexual Dysfunction
